# Prevalence of Heavy Menstrual Bleeding and Its Associated Factors Among Women Attending Kilimanjaro Christian Medical Centre In Northern Eastern, Tanzania: A Cross-Sectional Study

**DOI:** 10.24248/eahrj.v7i1.702

**Published:** 2023-07-12

**Authors:** Pendo Mussa Ibrahim, Ednah Loishiye Samwel

**Affiliations:** a Kilimanjaro Christian Medical University College.

## Abstract

**Background:**

Women of reproductive age experience a lower quality of life and considerable morbidity as a result of heavy menstrual bleeding. This issue needs to be addressed to achieve gender equality and permit women and girls to engage in a range of economic activities. In this study, we aimed to determine the prevalence and the most common factors associated with heavy menstrual bleeding.

**Methodology:**

Cross-sectional study was conducted at a zonal referral hospital in Northern Eastern, Tanzania. Data was extracted from women files who attended the hospital obstetrics and gynaecology clinic retrospectively. Data were analysed using Statistical Package for Social Sciences (SPSS) version 20.0. Descriptive statistics were used to summarize the data. A univariate logistic regression model was fitted to assess the strength of the association between heavy menstrual bleeding and exposure variables.

**Results:**

A total of 162 women aged 15-54 years were enrolled. The prevalence of heavy menstrual bleeding was found to be 24.1%. The following factors were found to be significantly associated with heavy menstrual bleeding; age range of 20-44 years (OR: 0.16;95% CI: 0.02-1.01), hormonal contraceptives (OR: 3.16; 95% CI: 1.15-8.69), having no clots on menstrual blood (OR: 0.19; 95% CI: 0.58-0.651), low haemoglobin level (OR: 5.61; 95% CI: 1.44-21.90), and uterine fibroid (OR: 0.35; 95% CI: 0.17-0.73).

**Conclusion:**

Despite the extreme measurements of Heavy Menstrual Bleeding (HMB) in this study, its prevalence remained high. To spread awareness of HMB and its consequences, we recommend screening the general public and offering health education initiatives.

## BACKGROUND

Menstruation in a woman is a normal physiological process. It is a monthly process when the woman is passing blood through the vagina from the reproductive tract, taking 3-8 days.^1^ Menstruation is a physiological process associated with several disorders in which heavy menstrual bleeding is among.^2^ Heavy menstrual bleeding can be defined as menstrual blood loss in a month of greater than 80mls.^3^ However, it can not only be measured objectively but also subjectively. The current and most common definition of heavy menstrual bleeding is menstrual blood loss that is so excessive it interferes woman's physical, social, emotional, or quality of life (QoL).^4^ It can also be considered when a woman passes large blood clots, need for double sanitary protection, needs for frequent changes of tampons and towels (meaning changes every 2 hours or less, or 12 sanitary items per period) and flooding through to clothes or bedding.^5^ Other objective measures of heavy menstrual bleeding involve the use of pictograms in estimating the volume of blood lost.^6^

The global prevalence of heavy menstrual bleeding has been demonstrated to differ. This prevalence also varies according to how HMB was measured. In America, the prevalence of heavy menstrual bleeding was 38.9 %.^7^ HMB prevalence was reported to be 27.2% in five European countries.^5^ Moreover, in developing countries, the prevalence of heavy menstrual bleeding is ranging from 8%-27%. There is a scarcity of data concerning the prevalence of heavy menstrual bleeding in African Countries.^8^ Heavy menstrual bleeding accounted for 57.4 % of all menstrual disorders assessed among adolescents in Nigeria.^9^ Heavy menstrual bleeding is associated with several factors and these don't necessarily imply causality but increase the risk of a woman experiencing heavy menstrual bleeding. These include age, parity, haemostatic defects, hormonal contraception, clots on menstrual blood, anaemia, and uterine fibroids.^9–12^ It has been documented that HMB affects the quality of life of many women of reproductive age as it interferes with their daily activities in terms of reducing the number of working hours, school attendance, and sexuality.^6^

Although heavy menstrual bleeding has never been documented to produce significant mortality, it causes significant morbidity due to anaemia.^4^ There is little knowledge and awareness among women and healthcare providers about the importance of reducing menstrual flow among women who are anaemic.^8^

To achieve gender equality and enable women and girls to participate in a variety of activities such as education, health care, a decent job, and representation in political and economic decision-making processes, this matter has to be addressed with an effective treatment plan. Considering the importance of this matter and the fact that data are scarce concerning the prevalence of heavy menstrual bleeding in East Africa simply because perhaps little attention has been given to this unmet area of reproductive health care for women, there is important to conduct this study. In this study, we aimed to determine, the prevalence of heavy menstrual bleeding and identify the most common factors associated with heavy menstrual bleeding among women attending Kilimanjaro Christian Medical Centre (KCMC), a tertiary referral hospital in North Eastern, Tanzania. This study will benefit other researchers by shedding light and creating a baseline for those who will be conducting studies on this subject.

## METHODOLOGY

### Study Design, Setting, and Population

We conducted this study from March to July 2016 at KCMC which is one of the referral zonal hospitals in Tanzania, serving mostly referral patient cases in the North-Eastern part of Tanzania. The minimum sample size was estimated using a formula expressed as sample size =Z^2^ (p)(1-p)/e2, where, Z = value (1.96 for 95 % confidence level).^13^ An average prevalence (P) of 7% for heavy menstrual bleeding reported from developing countries was used,^8^ and a minimal tolerable error at the 95% confidence level used was 0.05. The minimum estimated sample size was 110 non-pregnant patients who attended at obstetrics and gynaecology outpatient clinic of KCMC.

### Data Source and Collection Methods

**FIGURE 1:**
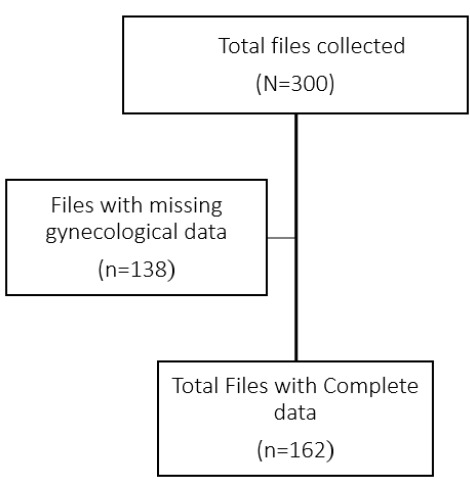
Flow Chart Showing Number of Screened and Selected Patients' Files

Data were extracted retrospectively from registered patient files of the women who attended the obstetrics and gynaecology clinic of KCMC in the year 2015 and filled in the data collection form. Only files involving non-gravid women with complete data were included. Additionally, a pre-testing of the data collection form was conducted before data collection. It involved selecting randomly 10 patient files to make sure the data needed for the study is available and not ambiguous. Also, pretesting was done for the validity and reliability of the data collection form. Non-probability sampling was employed in this study in which convenience sampling was done to select the files. The files with missing gynaecological or ineligible data were excluded. See flow chart figure 1 below.

### Study Variables

In this study, the outcome variable, heavy menstrual bleeding, was recognized when a woman self-reported using more than 5 fully soaked (wet) sanitary pads per day on any day of her menstrual period. Our operational definition was adopted with stringent measures to reduce research subjectivity and improve the study's clarity and reproducibility. Exposure variables included socio-demographic characteristics (age, residence/address, and occupation), reproductive and clinical characteristics (hormonal contraceptive use, Clots on menstruation, haemoglobin level, Platelet level, and Uterine fibroid).

### Statistical Analysis

Data were entered, cleaned, checked for completeness, and analysed using Statistical Package for Social Sciences (SPSS) software version 20. Descriptive statistics were used to summarise the data. Percentages and proportions were used to summarize categorical data while continuous data were summarized using the mean with their respective dispersion measure (standard deviation). The logistic regression model was used to determine the magnitude of the association. A univariate logistic regression model was fitted to obtain the crude estimates and a variable with a *P* value of <0.05 was considered statistically significant. The odds ratio with their 95% confidence interval was used to assess the strength of the association between heavy menstrual bleeding and exposure variables.

### Ethical Approval

The study received ethical approval from the ethics Committee of Kilimanjaro Christian Medical University College (KCMUCo). Permission was obtained from both the head of the gynaecology and obstetrics department and the medical record department to collect data.

## RESULTS

### Socio-demographic Characteristics among the Study Population

Participant ages ranged from 15-59 years with a mean age of 37.6 years (SD+ 9.98), 52.5% of participants were peasants while 53.7% resided in urban areas(Table 1).

**TABLE 1:** Socio-demographic Characteristics of Study Population (N=162)

variable	Number	%
Age		
Less than 19	8	4.9
20–44	106	65.4
Greater than 45	48	29.6
Occupation		
Peasant	85	52.5
Teacher	17	10.5
Student	18	11.1
Others	42	25.9
Residence		
Urban	87	53.7
Rural	75	46.3

### Reproductive Health and Clinical Characteristics of the Study Population

Among the 162 women, 73.5% (n=119) had ever had a child, 72.8% do not use hormonal contraceptives, 32.7% of women had a diagnosis of uterine fibroid, and 85.8% used sanitary pads as means of protection during their menstrual period. The mean and standard deviation result for the haemoglobin level of the women was 10.2g/dL and 2.9g/dL respectively. 53.7% of women were found to have a haemoglobin level of less than 11.9g/dL. Other characteristics of the participants are shown in Table 2.

**TABLE 2:** Reproductive Health and Clinical Characteristics of Study population (N=162)

variables	Number	%
Use of hormonal contraceptives		
Yes	44	27.2
No	118	72.8
Parity		
Never had a child	43	26.5
Ever had a child	119	73.5
Menstrual protection		
Sanitary pads	139	85.8
Cloth	22	13.6
Tampons	1	0.6
Hemoglobin level		
<8 g/dl	25	15.4
8g/dl-10.9 g/dl	53	32.7
11g/dl-11.9 g/dl	9	5.6
>11.9 g/dl	75	46.3
Platelet level*		
Less than 150,000/Ul	3	12.5
More than 150,000/Ul	21	87.5

Platelet level * = out of 162 files, 138 patient files missed data about platelet level

### Prevalence of Heavy Menstrual Bleeding

The prevalence of heavy menstrual bleeding was found to be 24.1% among women attending the obstetrics and gynaecological clinic of the KCMC hospital in North-Eastern Tanzania.

### Association of Participants' Socio-demographic Characteristics with Heavy Menstrual Bleeding

Age was found to be associated with heavy menstrual bleeding as a result of self-reported use of more than 5 sanitary pads per day. Women with ages ranging from 20-44 were having 84% fewer odds of having heavy menstrual bleeding compared to women aged 15-19 years. Other socio-demographic factors were assessed but found to be not significantly associated with heavy menstrual bleeding (Table 3).

**TABLE 3:** Association Between Socio-demographic Factors and Heavy Menstrual Bleeding (N=162)

variable	Number	n (%)	OR (95% CI)	P value
Age				
Less than 19 years	8	5 (12.82)	1	
20–44 years	106	21 (53.85)	0.16 (0.02–1.01)	0.02
> Than 45 years	48	13 (33.33)	0.26 (0.34–1.92)	
Occupation				
Peasant	85	14 (35.90)	0.38 (0.16–0.92)	
Teacher	17	4 (10.26)	0.57 (0.15–2.14)	
Students	18	7 (17.94)	0.69 (0.15–3.08)	0.08
Others	42	14 (35.90)	1	
Residence				
Urban	87	23 (58.97)	1	
Rural	75	16 (41.03)	0.76 (0.37–1.57)	0.47

### Association of Participants' Reproductive and Clinical Characteristics with Heavy Menstrual Bleeding

Significant factors that appear to be associated with heavy menstrual bleeding as a result of self-reported use of more than 5 fully soaked sanitary pads per day included the use of hormonal contraceptives, low haemoglobin level, presence of clots in menstrual blood and presence of uterine fibroid (Table 4).

**TABLE 4:** Association Between Reproductive and Clinical Characteristics of Study Population and Heavy Menstrual Bleeding (N=162)

Variable	Total	n (%)	OR (95% CI)	p-value
Use of hormonal contraceptive				
Yes	44	5 (12.82)	1	
No	118	34 (87.18)	3.157 (1.147–8.691)	0.023
Parity				
Never had a child	43	15 (38.46)	0.472 (0.218–1.019)	
Ever had a child	119	24 (61.54)	1	0.063
Menstrual protection				
Cloth	22	8 (20.51)	1	
Sanitary pads	139	31 (79.49)	0.738 (0.182–2.989)	0.305
Tampons	1	0 (0)	0	
Clots on menstruation				
Yes	12	7 (17.95)	0.194 (0.58–0.651)	
No	150	32 (82.05)	1	0.009
Hb level				
>11.9g/dl	75	11 (28.21)	1	
11–11.9 g/dl	9	0 (0)	2.28 (0.224–23.210)	
8–10.9 g/dl	53	17 (43.59)	1.806 (0.461–7.082)	
<8g/dl	25	11 (28.20)	5.401 (1.375–21.207)	0.016
Platelet level*				
<150,000/ul	3	3 (27.27)	1	
>150,000/ul	21	8 (72.73)	1.375 (0.958–1.975)	0.82
Uterine fibroid				
Yes	53	20 (51.28)	1	
No	109	19 (48.72)	0.348 (0.166–0.733)	0.006

* n=24

## DISCUSSION

The prevalence of heavy menstrual bleeding (HMB) was found to be high when a woman self-reported using more than 5 fully soaked sanitary pads per day. Women who do not use hormonal contraceptives, have no clots on menstrual blood, have severe anemia, or have uterine fibroids are more likely to self-report having heavy menstrual bleeding.

Depending on how it is assessed, HMB prevalence varies. Our prevalence values were remarkably within the self-reported prevalence rates for heavy menstrual bleeding in developing countries, which ranged from 8% to 27%.^8^ However, the prevalence we discovered was considerably higher than that of the Gambia study (4%).^14^

Moreover, this study's findings demonstrate that the prevalence is relatively lower than that of studies conducted in Europe and America, where the prevalence of heavy menstrual bleeding was 27.2% and 38.9% respectively.^5,7^ This result still supports the difference in prevalence that exists between developing and developed countries. This mismatch may be caused by the fact that menstruation subject is taboo in the African and Asian cultures.^15^ Because few women are aware of what constitutes a regular or atypical menstrual cycle, many of the affected women would opt for traditional remedies before eventually having medical attention.^15^ We can thus hypothesize that the prevalence of heavy menstrual bleeding could be higher in the general population of North Eastern, Tanzania.

In addition, a significant association between having a lower level of hemoglobin and heavy menstrual bleeding is similar to several studies done in East Africa, America, and Europe.^11,16^ Women in the current decade are more likely to experience more menstrual cycles due to advancements in fertility control that result in fewer cases of postpartum amenorrhea.^17^ Thus, there is a higher likelihood of getting anemia from an increasing number of menstrual periods in a woman's lifetime. Hence, women with HMB will more likely experience severe anemia. Anemia will thus affect women's physical well-being, which will lead to decreased engagement in social and economic activities.

Furthermore, the result of this study showed increased odds of having heavy menstrual bleeding in women who are not using hormonal contraceptives. Studies showing that hormonal contraceptives have an impact on lowering menstrual flow, menorrhagia, and the number of bleeding days can substantiate this.^18–20^ Hormonal imbalance secondary to multiple factors can cause HMB, thus hormonal contraception is one of the recommended first-line treatments to manage HMB.^21,22^ However, in this study, we couldn't differentiate the use of these medications as treatment for heavy menstrual bleeding or rather as a woman's choice of contraception.

Women presenting with clots in menstrual blood have fewer odds of having heavy menstrual bleeding compared to women who have no clots in menstrual blood. These findings were relatively similar to a European study which revealed that the presence of clots in menstrual blood does not contribute to the volume of blood loss. ^23^ In contrast to one of the American studies which indicate the positive association between the presence of clots in menstrual blood and heavy menstrual bleeding.^11^ In India, the decrease in clots in menstrual was associated with a decrease in heavy menstrual bleeding in women.^24^ The differences in results could be attributed due to the method used to assess HMB and study design. There are contrasting findings that need further research.

## CONCLUSION AND RECOMMENDATIONS

The present study yielded essential pieces of clinical reference information. Despite the stringent assessments of HMB in this study, there is a high prevalence of heavy menstrual bleeding in our region, 24.1%. Haemoglobin level less than 8g/dL, uterine fibroid, no clots on menstrual blood and use of hormonal contraceptives were associated with heavy menstrual bleeding. Moreover, Hormonal contraception continues to be protective against heavy menstrual bleeding. Lastly, reducing heavy menstrual bleeding will reduce the risk of anaemia and hence reduce the rate of morbidity among women. Thus from this Study's findings, we do recommend that health education programs should be established that help raise awareness of the consequences of heavy menstrual bleeding such as iron deficiency and anaemia, encourage women to seek assistance and thus improve their quality of life. Also, further studies are needed to explore more heavy menstrual bleeding in society so as to increase knowledge to help women improve their quality of life.
